# A phase 1b randomised, placebo-controlled trial of nabiximols cannabinoid oromucosal spray with temozolomide in patients with recurrent glioblastoma

**DOI:** 10.1038/s41416-021-01259-3

**Published:** 2021-02-24

**Authors:** Chris Twelves, Michael Sabel, Daniel Checketts, Sharon Miller, Bola Tayo, Maria Jove, Lucy Brazil, Susan C. Short, on behalf of the GWCA1208 study group, Catherine McBain, Brian Haylock, Paul Mulholland, Christopher Herbert, Allan James, Mohan Hingorani, Joerg Berrouschot, Rainer Fietkau, Jens Panse

**Affiliations:** 1 Leeds Institute of Medical Research at St James’s, University of Leeds, Leeds, UK; 2 Department of Oncology, Leeds Teaching Hospitals NHS Trust, St James’s University Hospital, Leeds, UK; 3 Department of Neurosurgery, Heinrich-Heine-University, Dusseldorf, Germany; 4 GW Research Ltd., Sovereign House, Vision Park, Chivers Way, Histon, Cambridge, UK; 5 Guy’s and St Thomas Hospitals Cancer Centre, Guys Hospital, Great Maze Pond, London, UK; 6 Department of Clinical Oncology, The Christie NHS Foundation Trust, Manchester, UK; 7 The Clatterbridge Cancer Centre NHS Foundation Trust, Bebington, Wirral UK; 8 University College Hospital, London, UK; 9 University Hospitals Bristol NHS Foundation Trust, Bristol, UK; 10 Cancer Research UK CTU, Beatson West of Scotland Cancer Centre, Glasgow, UK; 11 Hull and East Yorkshire NHS Trust, Castle Hill Hospital, Cottingham, UK; 12 Department of Neurology, Klinikum Altenburger Land GmbH, Altenburg, Germany; 13 Strahlenklinik, Universitätsklinikum Erlangen, Erlangen, Germany; 14 Hematology and Oncology Clinic, Universitätsklinikum Aachen, Aachen, Germany

**Keywords:** CNS cancer, Drug development, CNS cancer, Pharmaceutics

## Abstract

**Background:**

Preclinical data suggest some cannabinoids may exert antitumour effects against glioblastoma (GBM). Safety and preliminary efficacy of nabiximols oromucosal cannabinoid spray plus dose-intense temozolomide (DIT) was evaluated in patients with first recurrence of GBM.

**Methods:**

Part 1 was open-label and Part 2 was randomised, double-blind, and placebo-controlled. Both required individualised dose escalation. Patients received nabiximols (Part 1, *n* = 6; Part 2, *n* = 12) or placebo (Part 2 only, *n* = 9); maximum of 12 sprays/day with DIT for up to 12 months. Safety, efficacy, and temozolomide (TMZ) pharmacokinetics (PK) were monitored.

**Results:**

The most common treatment-emergent adverse events (TEAEs; both parts) were vomiting, dizziness, fatigue, nausea and headache. Most patients experienced TEAEs that were grade 2 or 3 (CTCAE). In Part 2, 33% of both nabiximols- and placebo-treated patients were progression-free at 6 months. Survival at 1 year was 83% for nabiximols- and 44% for placebo-treated patients (*p* = 0.042), although two patients died within the first 40 days of enrolment in the placebo arm. There were no apparent effects of nabiximols on TMZ PK.

**Conclusions:**

With personalised dosing, nabiximols had acceptable safety and tolerability with no drug–drug interaction identified. The observed survival differences support further exploration in an adequately powered randomised controlled trial.

**Clinical trial registration:**

ClinicalTrials.gov: Part 1– NCT01812603; Part 2– NCT01812616.

## Background

Glioblastoma (GBM) is the most common malignant primary brain tumour in adults, with an estimated incidence of 3.2 per 100,000, and 5-year survival rate of <6%.^1^ GBM is incurable, but current optimal therapy involves maximal debulking surgery followed by local high-dose radiotherapy and temozolomide (TMZ) chemotherapy.^2^ With this treatment median overall survival (OS) is 14.6 months for patients well enough to undergo treatment. Tumour recurrence occurs in almost all patients,^3^ usually 6–9 months after treatment. Following recurrence, median survival falls to 1.0–10.8 months,^4,5^ and there is no current standard of care for such patients.^6^

Despite preclinical and clinical efforts, improving outcomes for patients with GBM has proved challenging. Additional therapy with tumour-treating fields (TTF), which alternate electric fields at specific frequencies and intensities to disrupt mitosis in cancer cells, may impact OS,^7–9^ but has not become standard of care. In addition, subgroups of GBM patients with O^6^-methylguanine DNA methyltransferase (MGMT) promoter methylation have been shown to have a better prognosis and benefit from more aggressive initial therapy with TMZ and lomustine.^10–12^ As such, while recent advances in molecular pathology may allow for more individualised treatment in some patients, many patients with GBM do not benefit, and new treatments are urgently required.

Phytocannabinoids occur naturally in cannabis plants and have been used medicinally for centuries for a variety of purposes.^13^ Δ^9^-tetrahydrocannabinol (THC) is the major psychoactive constituent in cannabis, and cannabidiol (CBD) is the major non-psychoactive constituent; these are the most studied naturally occurring cannabinoids. In studies in animals and humans, THC can exert analgesic, antispasmodic, antitremor, anti-inflammatory, appetite stimulant and antiemetic properties.^14^ In studies in animals and humans, CBD can exert antiepileptic, neuroprotective, anti-inflammatory, antipsychotic, antidystonic and antiemetic effects.^15–19^

Unlike THC, CBD has no physiologically relevant effect on the cannabinoid receptors, CB_1_ and CB_2_, but targets other G protein-coupled receptors such as GPR12, GPR6, GPR3, GPR55 and 5-HT1A, as well as transient receptor potential vanilloid receptors, TRPV1 and TRPV2.^20–25^ THC is a partial CB_1_ and CB_2_ receptor agonist.^26^ Activation of CB_1_ and CB_2_ receptors exerts a variety of downstream signalling effects, with diverse consequences on cellular biology and functions.^27^

GBM tumours express both CB_1_ and CB_2_,^28^ with high-grade tumours expressing high levels of CB_2_. This altered expression of cannabinoid receptors in GBM led to the hypothesis that cannabinoids may exhibit antitumour effects. Numerous in vivo studies have found that administration of CBD and THC reduced tumour growth in animal models of glioma (reviewed by Rocha et al., 2014^29^). These effects are thought to be mediated by induction of cell death (via apoptosis or cytotoxic autophagy), inhibition of cell proliferation, and antiangiogenic effects (reviewed by Dimitru et al., 2018^30^). Specific to GBM, the combined administration of THC and TMZ exerts strong antitumoural effects in glioma xenografts, an effect maintained in tumours resistant to TMZ treatment.^31^ Furthermore, treatment with TMZ and submaximal doses of THC and CBD has strong antitumoural activity both in TMZ-sensitive and TMZ-resistant tumours.^31^

The first pilot trial to investigate the effects of intracranial THC (100 mg mL^−1^ in ethanol) in patients with recurrent GBM in 2006 showed a reduction in tumour cell proliferation in two of nine patients.^32^ However, this evidence of promising antineoplastic activity of THC and CBD in GBM has not yet been further assessed. Sativex^®^ ([nabiximols oromucosal spray] GW Research Ltd [GW], Cambridge, United Kingdom [UK]) is a complex botanical formulation containing THC, CBD, and additional cannabinoid and non-cannabinoid components. Nabiximols is approved for symptom improvement in patients with moderate to severe spasticity due to multiple sclerosis who have not responded adequately to other antispasticity medications in >25 countries but not the United States. In this trial, we investigate the safety and tolerability of nabiximols oromucosal spray in combination with dose-intense TMZ (DIT) in patients with recurrent GBM, as reflected by the frequency and severity of treatment-emergent adverse events (TEAEs: adverse events with onset or worsening after administration of the first dose of study drug, irrespective of relatedness to treatment). In addition, secondary efficacy outcomes, specifically patients’ progression-free survival at 6 months (PFS6) and OS at 1 year (i.e. the planned end of treatment), as well as the effects of nabiximols on TMZ and metabolite pharmacokinetics (PK), were assessed.

## Methods

### Compliance with ethical standards

This trial was conducted in accordance with International Council for Harmonisation Good Clinical Practice guidelines and ethical principles that have their origin in the Declaration of Helsinki. The protocol was approved by the relevant Institutional Review Board or Independent Ethics Committee at each site, and all patients provided written informed consent. The trial protocol is registered on the ClinicalTrials.gov website (Part 1: NCT01812603; Part 2: NCT01812616).

### Stopping rules

The trial could be terminated by the sponsor, primarily for safety, but also for other unanticipated reasons. Stopping rules for individual sites and patients are described in Supplemental Materials.

### Patients

Eligible patients were ≥18 years old with a histopathologically confirmed diagnosis of GBM (World Health Organization classification [version 2007^33^]) and evidence of first disease progression, following radiotherapy and first-line chemotherapy with TMZ. Patients had a ≥60% Karnofsky Performance Scale (KPS) status and, if taking steroids, were on a stable or reducing dose. All patients received standard of care (i.e. 6 weeks radiation therapy with concomitant TMZ then adjuvant TMZ).

### Trial design

This was a multisite, sequential, 2-part, Phase 1b trial. Patients were enrolled into Part 1 (open-label) or Part 2 (randomised, double-blind, placebo-controlled; 1:1 allocation), both of which followed the same schedule of visits and procedures (see Supplemental Fig. 1). Patients enrolled in Part 1 were not permitted to enter Part 2. The trial was conducted at 10 sites (7 UK; 3 Germany) between January 2014 and August 2016.

### Nabiximols and placebo dosing

Due to the high degree of interpatient variability in the PKs and pharmacodynamics of nabiximols,^34,35^ the dosing regimen was an individualised dose of 3–12 sprays/day, based on a dose-ranging trial that showed a favourable risk/benefit profile in patients with chronic pain.^36^ In both trial parts, treatment started with a single spray in the evening of Day 1, with gradual individualised titration of nabiximols or placebo (Part 2 only) by 1 additional spray/day, to a maximum dose based upon tolerability of up to 12 sprays/day (30 mg CBD and 32.4 mg THC). If a patient experienced unacceptable side effects during titration, the dose was reduced until the side effects resolved. Patients settled on a personalised maximum tolerated dose (MTD) within 14 days of their first dose of nabiximols or placebo and continued at that dose for the remainder of the trial. If patients experienced unacceptable side effects in the period following titration, they were advised to reduce their dose slightly until these resolved.

#### Part 1

Six patients participated in Part 1 (open-label) in two cohorts of three patients each. All received nabiximols and DIT with TEAEs recorded according to the common terminology criteria for adverse events (CTCAE version 4.03).

A safety review team (SRT) assessed the progress of Part 1 (open-label) of the trial to determine whether the second cohort in Part 1 and subsequently Part 2 could commence, as described in Supplemental Materials.

#### Part 2

Following approval from the SRT, 21 patients were randomised to receive DIT plus nabiximols or placebo using a 1:1 allocation ratio.

### Trial procedures

Patients commenced DIT on Day-7 (85.0 mg/m^2^ daily) and returned to trial sites on Day 1 when nabiximols (Parts 1 and 2) or placebo (Part 2 only), was dispensed. Patients titrated nabiximols/placebo as described above. Patients were instructed to take nabiximols or placebo at their personalised MTD for 1 year or until trial withdrawal; individual stopping rules are given in Supplemental Materials. DIT was administered orally for 13 cycles each of 28 days, i.e. up to 1 year with dosing on Days 1–21 of each cycle followed by 7 days off drug; the daily dose could be reduced to 70% and subsequently 50% if patients experienced DIT-related haematological TEAEs.

#### Pharmacokinetics

The effects of the addition of nabiximols to TMZ on the PK of TMZ and its metabolite, 4-amino-5-imidazole-carboxamide (AICA), were investigated. Blood sample collection, processing, and bioanalysis methods are described in Supplemental Materials.

PK parameters were derived by non-compartmental analysis using WinNonlin^®^ version 6.3 and included area under the plasma concentration-time curve to 6 h (AUC_0-6h_) and to the last timepoint (AUC_0-t_), maximum plasma concentration (C_max_), terminal (elimination) half-life (t_½_), time to maximum plasma concentration (t_max_), apparent oral clearance after oral administration (CL/F) and apparent volume of distribution after non-intravenous administration (V_z_/F).

### Randomisation and blinding

For Part 2 only (randomised), a 1:1 treatment allocation schedule with balanced randomly permuted blocks using a computer-based algorithm was produced independently; see Supplemental Materials for further information. Patients, investigators and the sponsor were blinded to the allocation and remained so until trial closure.

### Outcome measures

The primary objective of this trial was to investigate the tolerability and safety of nabiximols spray in patients with recurrent GBM, as indicated by the frequency and severity of TEAEs. Secondary objectives included investigating the preliminary efficacy of nabiximols compared with placebo, determined by PFS6 (magnetic resonance image [MRI] scans were performed for tumour assessment as per international consensus for both practice and trials via the revised assessment in neuro-oncology [RANO] criteria [additional MRI information can be found in Supplemental Materials])^3,37^ and survival at 1 year. Other secondary objectives were to determine any effects of nabiximols on TMZ PK.

### Unplanned (post-hoc) analyses

Since the trial was not designed to investigate survival differences, a post-hoc 2-year survival analysis was undertaken to further probe the effect of treatment in individual patients using the European Organisation for Research and Treatment of Cancer (EORTC) prognostic calculator of survival in patients with recurrent GBM.^38^ This calculated a predicted median (and range) OS for each trial patient that was compared with the actual outcome data. Patients were followed up for up to 27.5 months.

### Statistical analysis

#### Sample size

This was an exploratory Phase 1b trial with no formal sample size calculation. Part 1 (open-label) planned to enrol six patients and Part 2 (randomised) planned to enrol 20 patients.

#### Statistical methods

All hypotheses were tested at a 5% level of nominal statistical significance using a two-sided test. Due to the exploratory nature of this trial, no adjustments were made for multiple testing; Part 2 was randomised to ensure balance and limit bias in the safety comparisons. The safety analysis set was used for the analyses of all outcome parameters. Additional information can be found in Supplemental Materials.

## Results

### Disposition of patients

#### Part 1

Six patients enrolled in Part 1 (open-label), received nabiximols spray as planned and were included in the safety analysis.

**Fig. 1:**
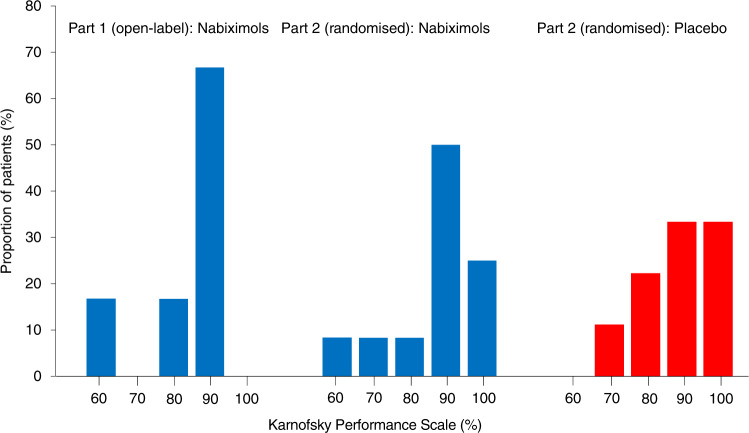
Proportion of patients in each KPS category at baseline. KPS Karnofsky Performance Scale.

The mean age of patients was 50.2 years (median 57, range: 28–67 years) and the mean time to diagnosis of recurrent GBM from initial diagnosis was 18.9 months (median 20.2 months, range: 8.6‒25.7 months). Mean time to patients entering the trial after the diagnosis of recurrence was 1 month (median 1.1 months, range: 0.5‒2.2 months). The median KPS at baseline was 90% (Supplemental Table 1); the proportion of patients in each KPS category is presented in Fig. 1.

**Fig. 2:**
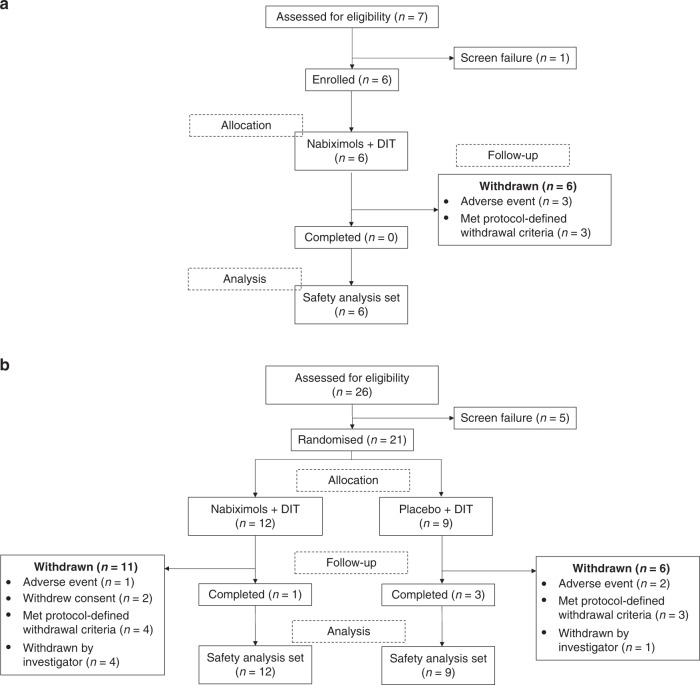
Disposition of patients enrolled. **a** Trial Part 1 (open-label). **b** Trial Part 2 (randomised). CBD cannabidiol, THC, Δ^9^-tetrahydrocannabinol.

Three patients (50.0%) came off study due to Grade 1 and 2 TEAEs (one due to lethargy, dizziness and fatigue, one due to nausea, diarrhoea and vomiting and one due to depressed mood), and three (50.0%) because of disease progression after an overall mean of 4.13 months for the six patients that came off study (Fig. 2a). There were no deaths during nabiximols treatment; three patients, however, subsequently died after coming off study.

#### Part 2

Twenty-one patients were randomised in Part 2 of the trial; 12 received nabiximols and nine received placebo, and all were included in the safety analysis.

The mean age was 57.8 years in both treatment groups (nabiximols median 59, range: 39–72 years; placebo median 57, range: 43–71 years). In the nabiximols and placebo treatment arms, 41.7% and 88.9% of patients were male, respectively. The mean time to diagnosis of recurrent GBM from initial diagnosis was 23.7 months (median 22.9 months, range: 3.1‒43.4 months; nabiximols group) and 21.7 months (median 19.6 months, range: 6.2‒55.7 months; placebo group). Mean time to patients entering the trial after the diagnosis of recurrence was 1.6 months (median 0.8 months, range: 0.4‒6.8 months; nabiximols group) and 0.8 months (median 0.8 months, range: 0.1‒2.0 months; placebo group). The median KPS at baseline was 90% for both patient groups (Supplemental Table 1); the proportion of patients in each KPS category is presented in Fig. 1.

In Part 2, 17 patients came off study (11 nabiximols and 6 placebo) and 4 patients (1 nabiximols and 3 placebo) completed the trial (~1 year of treatment) (Fig. 2b). Seven (41.2%) patients came off study due to disease progression (3 taking placebo; 4 taking nabiximols, 1 of whom also listed TMZ intolerance as a reason for withdrawal), 5 (29.4%) as a result of an investigator decision (1 considered too ill to continue study medication or chemotherapy [the patient was hospitalised due to a recurrent urinary infection which lasted for 3 months] and 4 due to disease progression), 3 (17.6%) due to TEAEs (1 due to concentration impairment and urinary incontinence and 2 due to disease progression) and 2 (11.8%) due to withdrawal of consent (1 due to a patient feeling too unwell to continue and 1 due to a patient feeling as though they were experiencing toxicity to the study medication and their concomitant medication [although no associated TEAEs were reported], requiring withdrawal per protocol).

Seven (33.3%) patients died during Part 2, 5 (55.6%) taking placebo and 2 (16.7%) taking nabiximols. Prior to death, all patients had come off study due to disease progression except for one patient taking nabiximols who came off study due to TEAEs.

### Drug exposure

#### Part 1

In Part 1, once titration had been completed and a stable personalised nabiximols dose established, patients took a mean of 6 sprays/day (range: 3.3–12 sprays/day). The mean duration of exposure was 16 weeks (median 15.1 weeks, range: 3.9–31 weeks). The mean dose of DIT was 146.6 mg (median 145.0 mg, range: 93.8–195.0 mg) or 77.8 mg/m^2^ (median 82.9 mg/m^2^, range: 49.2–85.4 mg/m^2^) relative to body surface area.

#### Part 2

Patients taking nabiximols administered a mean of 7.5 sprays/day after the titration period (range: 2.0–12 sprays/day); only one patient took fewer than the recommended minimum daily dose. The mean duration of exposure to nabiximols was 24.9 weeks (median 22.1 weeks, range: 7.1–50.9 weeks). The mean dose of DIT was 154.7 mg (median 153.9 mg, range: 117.5–185.0 mg) or 81.2 mg/m^2^ (median 83.3 mg/m^2^, range: 56.2–87.3 mg/m^2^) relative to body surface area.

Patients taking placebo administered a mean of 10 sprays/day after the titration period (range: 7.0–12 sprays/day) and the mean duration of exposure was 23.6 weeks (median 19.1 weeks, range: 1.9–51.3 weeks). The mean dose of DIT was 165.6 mg (median 170.0 mg, range: 125.0–195.0 mg), with a mean dose relative to body surface area of 81.1 mg/m^2^ (median 83.6 mg/m^2^, range: 63.3–86.5 mg/m^2^).

### Safety and tolerability

In both parts of the trial, the most frequent potentially clinically significant laboratory results were either low white blood cell counts or elevated liver enzymes. Abnormal laboratory results, reported as TEAEs, were consistent with the patients’ underlying disease and treatment with TMZ. There were no unexpected findings on physical examination, electrocardiogram, or vital signs. Compared with previous experience in nabiximols studies, no new safety concerns were identified.

#### Part 1

There were no fatalities during Part 1. Three (50.0%) patients experienced TEAEs, which led to discontinuation and three (50.0%) discontinued due to disease progression. TEAEs leading to discontinuation differed between patients and the maximal severity was CTCAE grade 2.

Table 1 reports TEAEs experienced by ≥2 patients and their maximal severity. Fatigue, dizziness, headache, vomiting and nausea were the most frequently observed TEAEs. Fatigue, dizziness and headache are commonly associated with GBM (fatigue and dizziness are also common side effects of nabiximols), and the incidence of nausea and vomiting is expected with chemotherapy and a recognised side effect of nabiximols. The serious AEs observed were neoplasm progression for one patient and intracranial haemorrhage (grade 1) and focal seizures (grade 1) for another; the patient who experienced intracranial haemorrhage had no changes to study medication at the time of the event.

**Table 1:** All-causality treatment-emergent adverse events reported in ≥2 patients (safety analysis set).

Part 1 (open-label)		
TEAE	Nabiximols *N* = 6	CTCAE Grade^a^
	Number of patients (%)	
Fatigue	4 (66.7)	2
Dizziness	3 (50.0)	1
Headache	3 (50.0)	2
Nausea	3 (50.0)	1
Vomiting	3 (50.0)	2
Vision blurred	2 (33.3)	2
Weight increased	2 (33.3)	1
Amnesia	2 (33.3)	1
Aphasia	2 (33.3)	1
Lethargy	2 (33.3)	2
Somnolence	2 (33.3)	1
Non-cardiac chest pain	2 (33.3)	3
Back pain	2 (33.3)	2

Part 2 (randomised)				
TEAE	Nabiximols *N* = 12	CTCAE Grade^a^	Placebo *N* = 9	CTCAE Grade^a^
	No. of patients (%)		No. of patients (%)	
Vomiting	9 (75.0)	1	1 (11.1)	1
Dizziness	8 (66.7)	2	2 (22.2)	1
Nausea	7 (58.3)	1	1 (11.1)	1
Fatigue	5 (41.7)	2	5 (55.6)	2
Headache	4 (33.3)	3	1 (11.1)	1
Constipation	4 (33.3)	3	0	N/A
Dry mouth	2 (16.7)	1	1 (11.1)	1
Thrombocytopenia	2 (16.7)	2	0	N/A
Feeling abnormal	2 (16.7)	1	0	N/A
Oedema peripheral	2 (16.7)	3	0	N/A
Cystitis	2 (16.7)	2	0	N/A
Urinary tract infection	2 (16.7)	4	1 (11.1)	1
Lethargy	2 (16.7)	1	0	N/A
Cough	2 (16.7)	1	0	N/A
Neoplasm progression	1 (8.3)	3	2 (22.2)	5
Urinary incontinence	0	N/A	2 (22.2)	2

*TEAE* treatment-emergent adverse event.

^a^Maximal severity in any patient who experienced the TEAE.

#### Part 2

Two patients (22.2%) in the placebo group and none in the nabiximols group died of disease progression by the time of the last planned follow-up.

The rates of TEAEs leading to discontinuation were similar in both treatment groups (two patients in each group [16.7% nabiximols vs. 22.2% placebo]). One patient in each group discontinued due to disease progression. The TEAEs leading to withdrawal in the remaining two patients were disturbance in attention, vomiting, ataxia and urinary incontinence for the placebo patient and urinary tract infection for the nabiximols patient.

Table 1 reports TEAEs experienced by ≥2 patients and their maximal severity. The incidence of TEAEs was similar in both treatment arms. Vomiting, dizziness, nausea and fatigue were the most frequently observed TEAEs. Patients taking nabiximols reported more severe TEAEs and had a higher incidence of serious TEAEs. Four patients taking nabiximols experienced serious TEAEs as follows: urinary tract infection for one patient, lower respiratory tract infection and anaemia for another, campylobacter gastroenteritis and asthenia for a third, and faecaloma for the fourth patient. Two patients in the placebo group experienced serious TEAEs as follows: neoplasm progression and pulmonary embolism for the first patient and neoplasm progression for the second patient.

Supplemental Table 2 presents the maximum toxicities of TEAEs experienced by each patient and any action taken as a result.

### Exploratory efficacy

#### Progression-free survival at 6 months

In Part 1, one (16.7%) patient was classified as progression-free at six months, four (66.7%) patients had progressed, and response status was unknown for one (16.7%) patient.

In Part 2, in the nabiximols group, four (33.3%) patients were classified as progression-free at six months, seven (58.3%) patients had progressed, and response status was unknown for one (8.3%) patient. In the placebo group, three (33.3%) patients were classified as progression-free at six months, five (55.6%) patients had progressed, and response status was unknown for one (11.1%) patient.

#### 1-year survival analysis

In Part 1, at 1 year three of six (50.0%) patients taking nabiximols were alive.

**Fig. 3:**
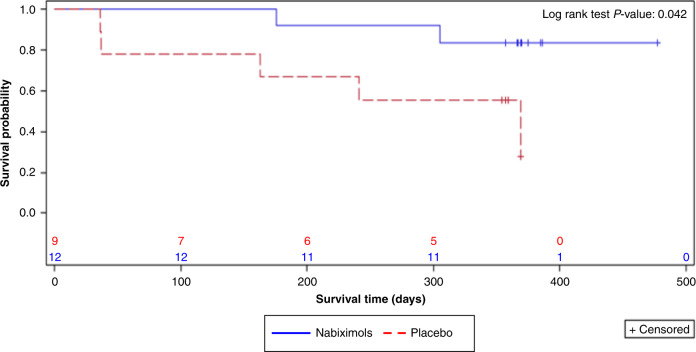
Kaplan–Meier survival curves (randomised safety analysis set). Patients who did not die during the 1-year analysis period were censored and marked by a +. If a patient was alive at the end of treatment, then the end of treatment visit was the date at which they were censored. Otherwise if a patient withdrew, they were censored at the date of the survival status review. If a patient was lost to follow-up then they were censored at the last known visit date. The number of patients at risk at a given timepoint was the number still alive or who had not been censored.

In Part 2, at 1 year 10 of 12 (83.3%) patients taking nabiximols were alive vs. four of nine (44.4%) patients taking placebo; although the trial was not statistically powered to compare OS, this difference in 1-year survival rate favouring nabiximols achieved nominal statistical significance (*p* = 0.042, log-rank test; Fig. 3).

#### Post-hoc 2-year survival analyses

Two-year survival data from elective follow-up of all patients (Parts 1 and 2) for a maximum of 27.5 months outside the protocol were also available, with OS at 2 years of 50% for patients treated with nabiximols and 22% for those treated with placebo (nominal *p* = 0.134, log-rank test). Median OS was estimated at 21.8 months (95% confidence interval [CI]: 10.0, not calculable [NC] as not enough events) for the nabiximols group and 12.1 months (95% CI: 1.18, NC) for the placebo group.

Post-hoc analysis using the EORTC prognostic calculator of survival for patients with recurrent GBM was used to calculate a predicted median (range) OS for each trial patient at enrolment that was then compared with the actual outcome for all patients based upon their 1-year survival (Supplemental Table 3 [Part 2]; Supplemental Table 4 [Part 1]).

In Part 2 of the trial, 10 (83.3%) patients taking nabiximols had OS that exceeded their EORTC-predicted median survival time; seven (58.3%) exceeded the upper range of their EORTC-predicted median survival time (Supplemental Table 3). Only one (8.3%) patient taking nabiximols survived for less than their lowest range predicted survival time.

In contrast, only three (33.3%) patients in the placebo group had OS that exceeded their EORTC-predicted median survival, one (11.1%) patient had OS as per their EORTC-predicted median survival, and only one (11.1%) patient exceeded the upper range of their EORTC-predicted median survival time. One (11.1%) patient was still alive after 25.5 months, so it was unknown whether they would exceed the upper range predicted (38.9 months) during extended follow-up. Two (22.2%) patients taking placebo who died during the trial had OS that fell within their predicted ranges and two (22.2%) patients had an OS time that fell short of the lowest time predicted. One (11.1%) patient, who discontinued the trial due to rapid progression and later died, could not have a predicted survival time defined by the EORTC tool (Supplemental Table 3).

### Pharmacokinetics

Patients excluded from the PK analysis are described in Supplemental Materials. In Parts 1 and 2, there was no effect on TMZ or AICA exposure (C_max_ and AUC_0-t_), or t_max_ when patients took DIT alone (Day 1) compared to co-administration with nabiximols (Day 36) (Supplemental Table 5 and Supplemental Table 6, respectively).

Statistical analysis was performed for Part 2 only. For TMZ, the ratio of geometric least squares (LS) means (95% CI) between the nabiximols and placebo groups for change in TMZ neared 1 for all exposure parameters, and was 1.02 (0.78, 1.33) for C_max_, 0.95 (0.82, 1.11) for AUC_0-6h_ and 0.91 (0.78, 1.05) AUC_0-t_.

For AICA, the ratio of geometric LS means (95% CI) between the nabiximols and placebo groups for change in TMZ was slightly greater than 1 for all but AUC_0-t_, and was 1.66 (0.70, 3.89) for C_max_, 1.21 (0.75, 1.95) for AUC_0-6h_ and 1.00 (0.64, 1.57) for AUC_0-t_.

## Discussion

This Phase 1b trial aimed to assess primarily the safety and tolerability and secondarily the efficacy of nabiximols in patients with recurrent GBM. In the absence of an accepted standard of care for this population and no known conventional dosing regimen for DIT, a DIT regimen of 85 mg/m^2^/day was chosen as the backbone chemotherapy since resistance to temozolomide is partially mediated by MGMT, and MGMT may be depleted by prolonged temozolomide administration. A DIT regimen may, therefore, overcome resistance^39^ and was considered an optimal comparator. Results showed the feasibility of a personalised dosing regimen of nabiximols in combination with DIT in patients with recurrent GBM, as per the primary outcome measures informing safety and tolerability. Additionally, the secondary outcomes of efficacy, and the effect of nabiximols on TMZ PK were assessed. Although both Parts 1 and 2 of the trial suggested increased efficacy, as defined by survival time, in patients treated with adjuvant nabiximols, any conclusions on efficacy are limited by the small sample size and potentially confounding factors that may differ between cohorts. PK outcomes suggested there was no significant effect of nabiximols on the systemic exposure of TMZ when administered as part of DIT.

There is a growing body of preclinical research that supports the antitumour activity of cannabinoids, including THC and CBD.^29,30^ Treatment with TMZ and submaximal doses of THC and CBD produced a strong antitumoural action in both TMZ-sensitive and TMZ-resistant GBM xenografts.^31^ The first pilot trial to evaluate the safety and efficacy of THC administered intratumourally in nine patients with recurrent GBM showed a good safety profile and possible antiproliferative effects of THC in two patients; however, effects on survival were unclear.^32^ Cerebral oedema was experienced by all patients in that trial in the early postoperative period following craniotomy. Additionally, there were mild, transient episodes of bulimia, hypothermia and euphoria in a single patient.^32^

The primary aim of the present trial was to analyse the safety and tolerability of personalised dosing of nabiximols oromucosal spray when co-administered with DIT. It should be noted that the safety findings from this study might not translate to a less fit patient population. The incidence of TEAEs in this trial was high in both nabiximols- and placebo-treated patients, for reasons discussed above. In Part 1 (open-label), most TEAEs were mild (CTCAE grade 1) or moderate (grade 2), the most frequently reported being fatigue, dizziness, headache, and vomiting. There were no grade 4 TEAEs.

In Part 2 (randomised), the incidence of TEAEs was higher in the nabiximols group than the placebo group, and those taking nabiximols reported more severe TEAEs and had a higher incidence of serious TEAEs. The two deaths during the trial occurred in the placebo group and were due to disease progression. This should be considered in the context of the sample overall sample size (*n* = 21) of Part 2 of the trial, with 12 patients receiving nabiximols and 9 placebo.

The incidence of TEAEs in this trial was higher than in previous Phase 3 randomised clinical trials of nabiximols as an adjunct analgesic in patients with advanced cancer (68‒72% in nabiximols-treated patients and 64‒66% in placebo-treated patients^34,35^). Given the nature of the present indication, many neurological and other TEAEs were considered related to the patient’s underlying GBM. Furthermore, the duration of treatment in the current trial was 1 year compared with 5‒7 weeks in previous Phase 3 trials.^34,35^ Finally, patients in the current trial received concomitant chemotherapy, which was precluded in previous Phase 3 trials. Therefore, the patient population and treatment regimen were different from other Phase 3 trials and may explain, in part, why there were more TEAEs in the current Phase 1b trial.

Specific attribution of TEAEs is complicated by the clinical presentation of GBM, the natural history of the disease, and concomitant administration of cytotoxic chemotherapy. Due to the palliative nature of GBM treatments, and the lack of therapeutic agents that are effective with a potentially acceptable TEAE frequency and severity profile, the results of this trial could support further clinical trials assessing the efficacy of adjuvant nabiximols in recurrent GBM.

The exploratory assessment of efficacy was based on patients’ PFS6 and OS at 1 year (i.e. the planned end of treatment). RANO assessments of MRI images showed no apparent effect of nabiximols on PFS6, with the same proportion of patients (33%) in the nabiximols and placebo groups progression-free at six months. In Part 1 (open-label nabiximols treatment), PFS6 was 16.7%. In both trial parts, PFS6 was higher than previously reported, whereas data pooled from 16 Phase 2 trials in 345 patients with recurrent GBM who received radiotherapy and various pharmaceutical therapies showed a PFS6 of only 9%.^36^ In Part 1 of the trial, OS at 1 year was found to be improved compared with previously published data, which put the death rate at ~80%.^36^ In Part 2 of the trial, a significant improvement in OS was observed at 1 year in patients taking nabiximols vs. placebo (83% vs. 44%; log-rank test, nominal *p* = 0.042). It is noteworthy that the 1-year OS rates across both treatment groups were higher than data pooled from several published trials of other agents, where OS at 1 year in 345 patients with recurrent GBM was only 14%.^36^ The high OS in our trial must be considered carefully as it may be explained in part by the small sample size.

On the basis of data from extended elective follow-up (to a maximum of 27.5 months) in the small numbers of patients originally enrolled in Part 2 (*n* = 21), median OS was estimated to be longer in the nabiximols group at 21.8 months (95% CI: 10.0, NC) vs. 12.1 months (95% CI: 1.18, NC) for placebo. This estimated length of OS is longer than previously reported in both patients newly diagnosed with GBM and those with recurrent GBM.^8,40^ A recent Phase 3 trial analysing the impact of TTFs in 695 patients newly diagnosed with GBM followed up patients for a median of 40 months (466 received TTFs plus TMZ; 229 received TMZ alone) and reported median OS of 20.9 months in the TTFs plus TMZ group and 16.0 months in the TMZ alone group.^40^ A Phase 3 trial analysing the impact of TTFs in 237 patients with recurrent GBM followed up patients for a median of 39 months (120 received TTF alone; 117 received chemotherapy alone) and reported median OS of 6.6 months in the TTF alone group versus 6.0 months in the chemotherapy alone group.^8^ We acknowledge that these data are from larger and more heterogenous populations than the study population in the present study. We did not collect treatment information after discontinuation of the study drug as part of this study so cannot discuss the potential impact of post-trial treatments on OS. However, there are no interventions known to significantly improve survival after discontinuing second-line treatment in this patient group. Moreover, as discussed above, this Phase 1b study had a small sample size and a possible imbalance in confounding factors between treatment groups may have impacted the interpretation of OS data. Also, of interest in the current trial was the trend for nabiximols-treated patients to have an OS which often exceeded the upper range of their EORTC-predicted survival (58% nabiximols patients vs. 11% placebo patients [Part 2]). While this model was not specifically developed on the basis of data from patients receiving DIT, there is no a priori reason to believe this invalidates the model for the prediction of survival in DIT-treated patients.

Of note, two patients in the placebo group died within 40 days of commencing treatment in Part 2 of the trial. It cannot be ruled out that the two patients concerned may have been predisposed to the premature death, e.g. by virtue of their tumour biology including unmethylated MGMT status. The lack of detailed tumour classification and documentation of prior therapy is a notable limitation of this trial, and the need for full characterisation in future trials, including MGMT methylation status, is recognised. Although there is no expectation of different outcomes between males and females, the considerable imbalance between males and females in this trial should be noted.

A final aim of this trial was to assess the effect of nabiximols on the PK of TMZ and AICA, to probe for a drug–drug interaction. There were no relevant effects of nabiximols on TMZ or AICA exposure parameters (C_max_ and AUC_0-t_), suggesting no clinically important impact of nabiximols on the PK of TMZ. The impact of TMZ on the PK of THC, CBD or their metabolites was not evaluated in this trial.

### Trial strengths and limitations

Key strengths of the trial were the individualised titration and personalised dosing of nabiximols and the randomised, placebo-controlled nature of Part 2. Without randomisation and placebo control, interpretation of the OS in patients treated with nabiximols would have been confounded.

The major limitation is that the number of patients enrolled in Part 2 of the trial was small, and with 21 patients enrolled across nine sites for Part 2, there was potential heterogeneity of practice and bias in patient selection between sites. In addition, 12 patients were assigned to nabiximols and nine to placebo during Part 2; this imbalance was due to randomisation being stratified by site. Therefore, although the trial was randomised, it is possible some imbalance between patients in the nabiximols and placebo arms existed, which might explain the deaths of two patients assigned to placebo within 40 days of enrolment. There was also no prespecified power calculation to determine the minimum number of patients required for adequate statistical power for efficacy endpoints. In addition, the trial used a non-conventional DIT dosing regimen. Finally, the EORTC tool to contextualise the OS data for individual patients was implemented after the trial was initiated. These data should, therefore, be interpreted with caution.

## Conclusion

Nabiximols spray appeared tolerable, and personalised dosing was feasible in this GBM patient population. No new safety concerns were identified, and there was no evidence to suggest an effect of nabiximols on the PK of TMZ. The observed survival differences should be interpreted with caution and justify further exploration in an adequately powered randomised controlled trial.

## Supplementary information

Supplementary material

## Data Availability

The trial protocol is registered on the ClinicalTrials.gov website (Part 1: NCT01812603; Part 2: NCT01812616). Supplemental Materials contain additional information about individual site and patient stopping rules, the safety review team, pharmacokinetic analysis, statistical methods, randomisation, patients excluded from the pharmacokinetic analysis, and Magnetic Resonance Imaging scans. Demographics and baseline characteristics are summarised in Supplemental Table 1. Maximum treatment-emergent adverse event toxicities for patients who experienced a grade 2 or higher treatment-emergent adverse event are summarised in Supplemental Table 2. EORTC-predicted vs. actual overall survival for the randomised element of the trial is summarised in Supplemental Table 3. EORTC-predicted vs. actual overall survival for the open-label element of the trial is summarised in Supplemental Table 4. Pharmacokinetic parameters for temozolomide and 4-amino-5-imidazole-carboxamide from the open-label element of the trial are summarised in Supplemental Table 5. Pharmacokinetic parameters for temozolomide and 4-amino-5-imidazole-carboxamide from the randomised element of the trial are summarised in Supplemental Table 6. The trial schema is supplied in Supplemental Fig. 1.
