# Supplementary material for: A phase 1b randomised, placebo-controlled trial of nabiximols cannabinoid oromucosal spray with temozolomide in patients with recurrent glioblastoma
Source: Br J Cancer. 2021 Feb 24;124(8):1379–87. doi: 10.1038/s41416-021-01259-3 (PMC8039032; doi:10.1038/s41416-021-01259-3)
Supplement: Supplementary file 1 — Supplementary material [file 41416_2021_1259_MOESM1_ESM.docx]

**Supplemental Materials**

Individual Site and Patient Stopping Rules

Individual sites could be terminated by the sponsor, Institutional Review Board/Independent Ethics Committee, or investigator due to lack of enrollment or non‑compliance with the protocol. Individual patients could be withdrawn from trial for any of the following reasons: pregnancy; a TEAE or protocol deviation that was considered to potentially compromise patient safety; lost to follow-up; administrative decision by the investigator, sponsor or regulatory authority; patient/legal representative withdrew consent; TMZ toxicity-related dose reduction for the third time; TMZ dose break (at investigator discretion); protocol non‑compliance; or disease progression.

Safety Review Team (Part 1)

The safety review team (SRT) primarily assessed adverse events (AEs), serious adverse events, and safety laboratory values. The SRT reviewed the safety profile of the first 3 patients to enter Part 1 (Cohort 1) once the third patient in Cohort 1 had reached Visit 5 (7 weeks after starting treatment). If >1 Cohort 1 patient experienced a grade 4 AE, then the SRT could implement changes to the treatment regimen. Cohort 2 then commenced (next 3 patients), and the SRT reviewed the safety profile of patients in Cohort 2 once the third patient reached Visit 5. If >1 Cohort 2 patient experienced a grade 4 AE, or >2 patients in total (Cohorts 1 and 2 combined) experienced a grade 4 AE, the SRT could implement changes to the treatment regimen or cease the trial entirely.

Pharmacokinetic Analysis

Blood samples were taken via an indwelling intravenous catheter or direct venipuncture on Day 1, 7 days into the first DIT cycle then again on Day 36, mid-way through the second DIT cycle and 14 days into dosing with nabiximols or placebo. On both days, 5 mL of blood was taken pre DIT dose, then at 20 and 40 minutes and 1, 1.5, 2, 4, and 6 hours post-dose. Blood samples collected for pharmacokinetic analysis were centrifuged for 5 min at 2000 g. The resultant plasma was stored in a freezer at −80°C.

PK analysis was undertaken by LGC (Cambridge, UK). Reference and internal standards for TMZ and AICA bioanalysis were supplied by Toronto Research Chemicals Inc (Toronto, Canada) and Sigma-Aldrich (Dorset, UK). TMZ and AICA samples were extracted from plasma by protein precipitation with formic acid and acetonitrile.

High-performance liquid chromatography (HPLC) with tandem mass spectrometry was performed using a Waters Acquity (Waters Corporation, Milford, MA, United States) system for HPLC and Sciex API4000 (AB Sciex Pte. Ltd, Singapore) system for mass spectrometry. Concentrations of TMZ and AICA were quantified in 100 µL human plasma samples. Chromatographic separations were performed on an Acquity BEH C18 column (internal diameter 1.7 μm, 2.1 × 100 mm). The selectivity of the HPLC method was checked by comparing chromatograms from blank plasma samples with chromatograms from spiked samples to ensure that there were no potential interfering peaks. Selectivity was ensured at the lower limit of quantification (LLOQ) for each analyte (10 ng/mL for TMZ and 20 ng/mL for AICA). Cross-interference between analytes was also assessed with samples spiked at the analytes’ upper level of quantification which was 10,000 ng/mL for TMZ and 2000 ng/mL for AICA.

The precision [coefficient of variation (%CV)] and accuracy [relative error (RE%)/mean % different (Bias%)] of the HPLC method was acceptable for all analytes was ≤ 15% (20% at the LLOQ). Recovery was 82.0–83.8% for TMZ and 77.0–78.9% for AICA.

Statistical Methods

Due to the exploratory nature of this trial, no adjustments were made for multiple testing; Part 2 was randomized to ensure balance and limit bias in the safety comparisons. All analyses were performed on the safety analysis set, which included all patients who received at least 1 dose of study product (nabiximols or placebo). All patients were analyzed according to trial part (Part 1 or 2) and study product (nabiximols or placebo) received.

The primary endpoint safety variables included incidence, type, and severity of all TEAEs, clinical laboratory assessments, and vital signs, for which summary statistics were produced.

The number and percentage of patients who were progression-free, not progression‑free, and unknown, were summarized (by Part 2 treatment group) and PFS6 for nabiximols vs. placebo compared using Fisher’s exact test.

Survival was assessed on Day 359 (1 year/the end of trial treatment) or trial withdrawal. Kaplan‑Meier survival curves by randomized treatment (nabiximols or placebo) were generated and median survival times with 95% confidence intervals (CIs) estimated. The difference in survival for nabiximols vs. placebo was compared using a log‑rank test. A post‑hoc, unplanned 2-year survival analysis was also conducted in the same manner. Due to the nature of this analysis, however, p-values should be considered nominal.

Also, in Part 2, analysis of the change from baseline (Day 1, TMZ only) to Day 36 (TMZ + nabiximols or placebo) in TMZ PK parameters was performed using analysis of covariance, with baseline values as a covariate and treatment as a factor.

Randomization

Patients were randomized using the next available allocation within the randomization schedule stratified by site. The randomization schedule was held centrally and not divulged to other persons involved in the trial until the database was locked.

Patients Excluded from the Pharmacokinetic Analysis

In Part 1, 2 patients were excluded from the Day 1 PK summary statistics; 1 was an outlier and 1 was under-dosed with TMZ. Three patients were excluded from the Day 36 PK summary statistics; 1 had no PK samples taken, 1 had PK collected outside of the specified nominal time points, and 1 had discontinued nabiximols due to TEAEs. In Part 2, 1 patient’s data were excluded from the Day 1 PK summary statistics due to non-compliance with the protocol (eligibility not re-checked, and dexamethasone prescribed on Day 1). Eight patients were excluded from the PK summary statistics on Day 36; 2 had stopped taking nabiximols due to TEAEs and 7 patients (including both already excluded due to TEAEs) had PK samples analyzed outside the defined period of stability.

MRI Scans

Magnetic resonance image (MRI) scans were performed for tumor assessment via the RANO criteria. Any changes to neurologic function and steroid dose were also taken into consideration. A baseline MRI scan was required to ensure that the patient satisfied the trial entry criteria. A trial-specific MRI scan was required at screening unless a standard clinical assessment MRI scan, less than 4 weeks old, was available at screening. The baseline MRI scan needed to demonstrate evidence of tumor progression for the patient to be eligible for the trial. For the duration of the trial all MRI scans were assessed using the same techniques, scanner and qualified technician as at the baseline assessment (where possible). Evidence of the scan and the RANO assessment was to be documented accordingly and be available for archiving at the end of the trial.

The collected information included:

- Presence of measurable disease (yes/no).
- Number of measurable lesions.
- Site(s).
- Largest diameter (mm).
- Cross sectional.
- Perpendicular.
- Product of cross sectional and perpendicular.
- Presence of non-measurable lesions (yes/no).
- Number of non-measurable lesions.
- RANO criteria assessment grade.

**Supplemental Tables**

**Supplemental Table 1**: Demographics and baseline characteristics (safety analysis set)

| **Parameter** | **Part 1** | **Part 2** | |
| --- | --- | --- | --- |
|  | **Nabiximols**  **N=6** | **Nabiximols N=12** | **Placebo N=9** |

|  | | | **Mean (SD)** | | |
| --- | --- | --- | --- | --- | --- |
| Age (years) | | 50.2 (17.21) | | 57.8 (10.76) | 57.8 (8.29) |
| KPS at baseline (continuous) (%)^a^ | | 90 (60–90) | | 90 (60–100) | 90 (70–100) |
| Time from diagnosis to trial entry (months) | | 1.12 (0.62) | | 1.57 (1.78) | 0.84 (0.57) |
| Time from initial to recurrent diagnosis (months)^b^ | | 18.9 (6.46) | | 23.7 (11.0) | 21.7 (15.1) |
|  | | **Number of Patients (%)** | | | |
| Sex | Male | 3 (50.0) | | 5 (41.7) | 8 (88.9) |
|  | Female | 3 (50.0) | | 7 (58.3) | 1 (11.1) |

KPS, Karnofsky Performance Status; SD, standard deviation.

^a^ Median and range.

^b^ Diagnosis refers to histopathologically confirmed diagnosis of recurrent GBM.

**Supplemental Table 2:** Part 1 (open-label) and Part 2 (randomized) maximum TEAE toxicities for patients who experienced a grade 2 or higher TEAE, including action taken with study medication (safety analysis set)

| **Patient** | **Maximum Grade** | | **TEAE MedDRA PT** | **Action taken with IMP** |
| --- | --- | --- | --- | --- |
| **Part 1 – Open-label** | | | | |
| **Nabiximols** | | | | |
| 022 | 3 | Pulmonary embolism | | None^1^ |
| 030 | 3 | Neoplasm progression | | None^2^ |
|  | 3 | Non-cardiac chest pain | | None^2^ |
| 009 | 2 | Depressed mood | | Stopped |
| 011 | 2 | Diarrhoea | | Stopped |
|  | 2 | Gastritis | | Dose reduced |
|  | 2 | Headache | | None |
|  | 2 | Hemiparesis | | None |
|  | 2 | Back pain | | None |
|  | 2 | Fatigue | | None |
| 010 | 2 | Vision blurred | | None |
|  | 2 | Gait disturbance | | None |
| 006 | 2 | Gastrooesophageal reflux disease | | None^3^ |
| **Part 2 - Randomized** | | | | |
| **Nabiximols** | | | | |
| 021 | NA^4^ | ‒ | | ‒ |
| 029 | 4 | Urinary tract infection | | Stopped |
| 024 | NA^4^ | ‒ | | ‒ |
| 015 | 4 | Anaemia | | Interrupted^5^ |
| 012 | 2 | Weight decreased | | None^6^ |
| 003 | 2 | Vomiting | | None |
|  | 2 | Constipation | | None |
| 017 | NA^4^ | ‒ | | ‒ |
| 001 | 2 | Rash | | None |
|  | 2 | Dizziness | | Interrupted |
|  | 2 | Fatigue | | Interrupted |
|  | 2 | Disorientation | | Interrupted |
|  | 2 | Campylobacter gastroenteritis | | None |
|  | 2 | Asthenia | | None |
| 032 | 3 | Fall | | Interrupted |
| 020 | 3 | Faecaloma | | None^7^ |
|  | 3 | Oedema peripheral | | None^7^ |
|  | 3 | Myopathy | | None^7^ |
|  | 3 | Constipation | | None^7^ |
| 007 | 3 | Neoplasm progression | | Stopped |
| 005 | 3 | Pain | | None |
| **Placebo** | | | | |
| 031 | 5 | Neoplasm progression | | Stopped |
| 014 | 2 | Urinary incontinence | | None |
|  | 2 | Faecal incontinence | | None |
|  | 2 | Fatigue | | None |
| 025 | 2 | Urinary tract infection | | None |
| 002 | 2 | Oropharyngeal pain | | None |
|  | 2 | Fatigue | | None |
| 018 | NA^4^ | ‒ | | ‒ |
| 008 | 5 | Neoplasm progression | | Stopped |
| 027 | NA^4^ | ‒ | | ‒ |
| 019 | NA^8^ | ‒ | | ‒ |
| 013 | 2 | Ataxia | | Stopped |
|  | 2 | Disturbance in attention | | Stopped |
|  | 2 | Urinary Incontinence | | Stopped |

IMP, investigational medicinal product; MedDRA, Medical Dictionary for Regulatory Activities; NA, not applicable; PT, preferred term; TEAE, treatment-emergent adverse event.

^1^ However, the patient had their nabiximols dose reduced and stopped due to milder TEAEs.

^2^ However, the patient had their nabiximols dose stopped due to milder TEAEs.

^3^ However, the patient had their nabiximols dose reduced due to a milder TEAE.

^4^ No grade 2 or above TEAEs. All patients with only grade 1 TEAEs had no action taken with their IMP.

^5^ However, the patient had their nabiximols dose interrupted due to milder TEAEs.

^6^ However, the patient had their nabiximols dose reduced due to milder TEAEs.

^7^ However, the patient had their nabiximols dose reduced and stopped due to milder TEAEs.

^8^ This patient did not experience any TEAEs during the trial.

**Supplemental Table 3:** Part 2 (randomized) overall survival; predicted by EORTC vs. actual (safety analysis set)

| **Patient** | **EORTC Predicted Median OS^a^**  **(months [range])** | **OS**^a,b^  **(months)** |
| --- | --- | --- |
| **Nabiximols** | | |
| 021 | 4.7 (3.61–5.88) | >12.0 |
| 029 | 7.92 (6.44–11.04) | >12.7 |
| 024 | 11.1 (8.71–16.99) | >12.7 |
| 015 | 7.92 (6.44–11.04) | >12.1 |
| 012 | 11.04 (7.72–18.2) | >11.7 |
| 017 | 7.13 (5.59–10.74) | >15.7 |
| 003 | 11.1 (8.71–16.99) | 5.8 |
| 001 | 7.92 (6.44–11.04) | >12.1 |
| 032 | 8.38 (6.21–12.88) | >12.2 |
| 020 | 6.28 (5.19–9.49) | 10.0 |
| 007 | 18.2 (11.37–38.87) | >12.3 |
| 005 | 4.7 (3.61–5.88) | >12.1 |
| **Placebo** | | |
| 031 | 5.72 (4.9–6.87) | 1.2 |
| 014 | 11.04 (7.72–18.2) | 12.1 |
| 025 | 7.92 (6.44–11.04) | 7.9 |
| 002 | 11.1 (8.71–16.99) | >11.6 |
| 018 | 18.2 (11.37–38.87) | >11.7 |
| 027 | 18.2 (11.37–38.87) | >11.8 |
| 008 | 7.92 (6.44–11.04) | 1.2 |
| 019 | Undefined | 5.4 |
| 013 | 5.75 (4.44–7.72) | >12.1 |

EORTC, European Organization for Research and Treatment of Cancer; OS, overall survival.

Note: ‘>’ denotes patients that remained alive at the last assessment point.

^a^ Survival in months; ^b^ Based upon 1‑year survival data.

**Supplemental Table 4:** Part 1 (open-label) overall survival; predicted by EORTC vs. actual (safety analysis set)

| **Patient** | **EORTC predicted median OS (range)**^a^ | **OS**^a,b^ |
| --- | --- | --- |
| 022 | 6.28 (5.19–9.49) | >12.0 |
| 009 | 7.92 (6.44–11.04) | >12.0 |
| 030 | 3.09 (2.46–4.37) | 10.2 |
| 011 | 7.92 (6.44–11.04) | >19.0 |
| 010 | 7.92 (6.44–11.04) | 8.2 |
| 006 | 5.72 (4.9–6.87) | 7.7 |

EORTC, European Organisation for Research and Treatment of Cancer; OS, overall survival.

^a^ Survival in months.

^b^ Based upon 1‑year survival data.

**Supplemental Table 5:** Part 1 (open-label) PK parameters for TMZ and AICA (safety analysis set)

| **Parameter** | **Unit** | **DIT Alone (Day 1)** | **DIT + Nabiximols (Day 36)** |
| --- | --- | --- | --- |
| **TMZ** | | | |
| C_max_^a^ | ng/mL | 5690 (34.0)^c^ | 4810 (24.5)^d^ |
| t_max_^b^ | h | 0.75 (0.7–1.0)^c^ | 0.65 (0.60–0.70)^d^ |
| t_½_^b^ | h | 1.7 (1.6–1.8)^c^ | 1.8 (1.7–1.8)^d^ |
| AUC_0–6h_^a^ | h·ng/mL | 13400 (13.0)^c^ | 12100 (8.24)^d^ |
| AUC_0–t_^a^ | h·ng/mL | 13400 (13.0)^c^ | 12100 (8.24)^d^ |
| CL/F^a^ | mL/min/m^2^ | 95.0 (13.5)^c^ | 104 (6.06)^d^ |
| V_z_/F^a^ | L/m^2^ | 14.1 (12.3)^c^ | 15.9 (12.5)^d^ |
| **AICA** | | | |
| C_max_^a^ | ng/mL | 572 (13.9)^c^ | 551 (20.6)^d^ |
| t_max_^b^ | h | 2.0 (1.5−2.0)^c^ | 2.1 (2.0–2.1)^d^ |
| t_½_^b^ | h | 2.5 (2.5−2.5)^e^ | NC^f^ |
| AUC_0–6h_^a^ | h·ng/mL | 2300 (13.8)^c^ | 2230 (12.0)^d^ |
| AUC_0–t_^a^ | h·ng/mL | 2300 (13.8)^c^ | 2230 (12.0)^d^ |

AICA, 4-amino-5-imidazole-carboxamide; AUC_0-6h_, area under the plasma concentration-time curve from time zero to 6 hours; AUC_0-t_, area under the plasma concentration-time curve from time zero to the last time point; CL/F, apparent oral clearance; C_max_, maximum plasma concentration; CV%, coefficient of variation; NC, not calculable; t_½_, terminal (elimination) half-life; t_max_, time to maximum plasma concentration; TMZ, temozolomide chemotherapy; V_z_/F, apparent volume of distribution.

^a^ Geometric mean (CV%).

^b^ Median (range).

^c^ n=4; ^d^ n=2; ^e^ n=1; ^f^ n=0.

**Supplemental Table 6:** Part 2 (randomized) summary of TMZ and AICA PK parameters (safety analysis set)

| **Parameter** | **Unit** | **Nabiximols** | | **Placebo** | |
| --- | --- | --- | --- | --- | --- |
|  |  | **DIT Alone (Day 1)** | **DIT + Nabiximols**  **(Day 36)** | **DIT Alone (Day 1)** | **DIT + Placebo**  **(Day 36)** |
| **TMZ (geometric mean [CV%])** | | | | | |
| C_max_ | ng/mL | 4540 (40.1)^b^ | 4340 (25.1)^b^ | 4230 (34.2)^c^ | 4690 (28.8)^g^ |
| t_max_^a^ | h | 1.2 (0.3–2.0)^b^ | 0.9 (0.3–2.0)^b^ | 1.1 (0.7–2.0)^c^ | 1.3 (0.7–1.6)^g^ |
| t_½_^a^ | h | 1.6 (1.5–1.9)^c^ | 1.9 (1.7–2.4)^d^ | 1.9 (1.7–2.0)^f^ | 1.9 (1.7–2.6)^g^ |
| AUC_0–6h_ | h·ng/mL | 13100 (19.8)^b^ | 12600 (12.3)^e^ | 11900 (25.1)^d^ | 13900 (11.9)^g^ |
| AUC_0–t_ | h·ng/mL | 13100 (19.8)^b^ | 12300 (13.5)^b^ | 12000 (22.5)^c^ | 13900 (11.9)^g^ |
| CL/F | mL/min/m^2^ | 92.7 (14.5)^c^ | 99.9 (13)^d^ | 90.8 (24.5)^f^ | 85.1 (12.6)^g^ |
| V_z_/F | L/m^2^ | 13.0 (19.4)^c^ | 16.3 (26.3)^d^ | 14.5 (15.0)^f^ | 14.7 (17.3)^g^ |
| **AICA (geometric mean [CV%])** | | | | | |
| C_max_ | ng/mL | 584 (20.3)^b^ | 701 (65.0)^b^ | 483 (20.6)^c^ | 450 (20.6)^g^ |
| t_max_^a^ | h | 2.0 (0.7–4.0)^b^ | 2.2 (0.67–4.1)^b^ | 2.0 (1.5–4.0)^c^ | 2.0 (2.0–4.0)^g^ |
| t_½_^a^ | h | NC^h^ | NC^h^ | NC^h^ | NC^h^ |
| AUC_0–6h_ | h·ng/mL | 2410 (14.4)^e^ | 2560 (33.1)^c^ | 1880 (16.2)^d^ | 1970 (15.7)^g^ |
| AUC_0–t_ | h·ng/mL | 2360 (14.5)^b^ | 2370 (42.5)^b^ | 1800 (17.7)^c^ | 1970 (15.7)^g^ |

AICA, 4-amino-5-imidazole-carboxamide; AUC_0-6h_, area under the plasma concentration-time curve from time zero to 6 hours; AUC_0-t_, area under the plasma concentration-time curve from time zero to the last time point; CL/F, apparent oral clearance; C_max_, maximum plasma concentration; CV%, coefficient of variation; NC, not calculable; t_½_, terminal (elimination) half-life; t_max_, time to maximum plasma concentration; TMZ, temozolomide chemotherapy; V_z_/F, apparent volume of distribution.

^a^ Median (range).

^b^ n=8; ^c^ n=6; ^d^ n=5; ^e^ n=7; ^f^ n=3; ^g^ n=4; ^h^ n=0.

**Supplemental Figure**

**Supplemental Figure 1:** Trial schema. Participation in Part 1 (open-label) or Part 2 (randomized) was identical in terms of the patient visit schedule and procedures undertaken

Visit 18, Day 386

Visit 1

Visit 3, Day 22

Visit 17, Day 358

Visit 2, Day 1

F

U

L

L

A

S

S

E

S

S

M

E

N

T

V

I

S

I

T

E

N

D

O

F

T

R

E

A

T

M

E

N

T

IMP

T

I
T
R
A
T
I
O
N

*During Treatment Period*

*Visits 4–7 every 14 days*

*Visits 7–17 every 28 days*

FO

L

L

O

W

U

P

S

C

R

E

E

N

I

N

G

Nabiximols + DIT

Open-label phase (6 patients)

Nabiximols + DIT

Randomized phase (12 patients)

*(Nabiximols/placebo allocation at Visit 2)*

Placebo + DIT

Randomized phase (9 patients)

*7–14 days*

*21 days*

*336 days*

*28 days*

*Stable IMP + DIT
(for 1 year or until withdrawal)*

*First DIT dose 7 days prior to Visit 2*

DIT, dose intense temozolomide; IMP, investigational medicinal product.
